# Dynamic hyper-editing underlies temperature adaptation in *Drosophila*

**DOI:** 10.1371/journal.pgen.1006931

**Published:** 2017-07-26

**Authors:** Ilana Buchumenski, Osnat Bartok, Reut Ashwal-Fluss, Varun Pandey, Hagit T. Porath, Erez Y. Levanon, Sebastian Kadener

**Affiliations:** 1 The Mina and Everard Goodman Faculty of Life Sciences, Bar Ilan University, Ramat Gan, Israel; 2 Biological Chemistry Department, Silberman Institute of Life Sciences, The Hebrew University of Jerusalem, Jerusalem, Israel; Stanford University, UNITED STATES

## Abstract

In *Drosophila*, A-to-I editing is prevalent in the brain, and mutations in the editing enzyme ADAR correlate with specific behavioral defects. Here we demonstrate a role for ADAR in behavioral temperature adaptation in *Drosophila*. Although there is a higher level of editing at lower temperatures, at 29°C more sites are edited. These sites are less evolutionarily conserved, more disperse, less likely to be involved in secondary structures, and more likely to be located in exons. Interestingly, hypomorph mutants for ADAR display a weaker transcriptional response to temperature changes than wild-type flies and a highly abnormal behavioral response upon temperature increase. In sum, our data shows that ADAR is essential for proper temperature adaptation, a key behavior trait that is essential for survival of flies in the wild. Moreover, our results suggest a more general role of ADAR in regulating RNA secondary structures *in vivo*.

## Author summary

In this work, we study one of the most abundant, yet poorly characterized genomic phenomena that has the potential to change the basic biological dogma–RNA editing, which creates transcriptome diversity by transforming adenosine into guanosine in RNA sequences. Such alteration, which is performed by ADAR family of deaminases, does not damage the original genomic version, and can be revised when circumstances change. Our analysis demonstrates that ADAR plays an important role in temperature adaptation by sensing and acting globally on RNA secondary structure. We suggest that ADAR has evolved to be highly efficient at cold temperatures, where RNA secondary structure is more prevalent. On the contrary, at high temperatures, where the secondary structure is more labile, ADAR may have negative effects, as it increases the chance of substitution in exonic sequences. Moreover, we observed behavioral defects in the ADAR hypomorphs at high temperatures.

## Introduction

Control of RNA function is essential for all living organisms. Gene expression is regulated at the transcriptional, co-transcriptional and post-transcriptional levels. Regulation at the post-transcriptional level is achieved by alternative pre-mRNA processing, regulation of RNA turnover, control of RNA localization or translation. Post-transcriptional RNA modifications also serve to regulate the functions of specific coding or non-coding RNA molecules without any impact at the DNA level[1].

A prevalent RNA modification is A-to-I editing. The reaction is catalyzed by the ADAR family of enzymes[2]. ADAR stands for “adenosine deaminase acting on RNA”. These enzymes deaminate the nucleotide adenosine (A), converting it to inosine (I)[3,4]. In RNAs that encode proteins, this modification can lead to an amino-acid substitution, as (I) is recognized as guanosine (G) by the ribosome[5]. Until recently, RNA editing events were considered rare; however, since the advent of next generation sequencing technologies, millions of editing sites have been identified in metazoan transcriptomes[6–8] including that of *Drosophila melanogaster*[9–12]. Hundreds of the editing sites are located in fly coding sequences, and many of them alter protein sequence, thus expanding the proteome diversity and increasing genomic flexibility. A-to-I editing can also influence the protein repertoire by creating or destroying splice sites of coding exons[13,14]. Most of the editing events occur in non-coding regions including introns and 3’ and 5’ untranslated regions (UTRs). Some of those editing events regulate RNA degradation[15] and microRNA (miRNA) function[16].

Identifying novel editing sites is not easy, since A-to-G mismatches can occur due to somatic mutations, genomic polymorphisms, and sequencing or alignment errors[17]. Traditionally, RNA editing events are identified by aligning the RNA-seq data to the genome. Recently, we developed an approach[12] that can detect RNA editing events with high specificity without the need for matched DNA. Our algorithm relies on the observation that ADAR enzymes tend to edit their substrates at multiple sites, resulting in dense clusters of editing events. Those heavily edited regions, which are usually missed by other editing detection approaches, contain reliable A-to-I events. Using this approach we revealed that A-to-I transition occurs frequently in *Drosophila*, especially in the brain[12].

RNA editing is critical to the function of the nervous system. Indeed, A-to-I editing re-coding events are found mainly in neuronal genes, and functional effects of editing on ion channels and G-protein coupled receptors have been demonstrated[18–21]. Editing is important for normal brain function in *Drosophila*[22] as mutants lacking the ADAR enzyme exhibit behavior alterations including defects in flight, motor control, and mating[22]. There are three ADAR genes in mammals (encoding ADAR1, ADAR2, and ADAR3), but *Drosophila melanogaster* has only a single ADAR protein (dADAR), an ortholog of the mammalian ADAR2. ADAR1 was lost during the evolution of insects[23].

Interestingly, most A-to-I editing events in flies do not seem to impact the coding sequence and do not alter directly gene expression by altering splicing or miRNA binding sites, leading researchers to postulate that most RNA editing events are non-functional[24]. However, ADAR has also been shown to regulate the unwinding of double-stranded RNAs[25]. ADAR displays strong specificity for long double-stranded RNA structures, with little sequence preference. In mammals, the disruption of endogenous secondary structures by ADAR is required to prevent activation of the cytosolic innate immune system[26–28], suggesting a role of A-to-I editing in general sensing and disruption of secondary structures.

Therefore, it is not surprising that ADAR activity appears to be modulated in situations in which RNA secondary structure is altered. One of these situations is temperature change, in particular in poikilotherm organisms like *Drosophila* that do not regulate internal temperature. Indeed, temperature is a strong regulator of fly physiology and behavior[29]. The pattern of locomotor activity is immediately changed when flies are transferred between temperatures[29]. Interestingly, recent work demonstrates that ADAR activity in *Drosophila* is strongly influenced by temperature[30]. At a subset of well-characterized editing sites, editing levels were significantly higher at 15°C than at 35°C. This is likely due to the lower stability of the structures surrounding the editing sites as well as to a strong inhibition of ADAR activity at 35°C. The effect on ADAR activity is achieved both by downregulation of *adar* expression and by a decrease in ADAR activity. Interestingly, at higher temperatures *adar* mRNA is auto-edited, resulting in a protein with diminished editing activity. The fact that ADAR activity is tightly regulated by temperature suggests that A-to-I editing is important for the adaptation of flies to temperature changes[31], perhaps through regulation of RNA secondary structure.

Here we investigate the role of ADAR in temperature adaptation in *Drosophila*. We first fully characterized the A-to-I editing landscape in heads of flies adapted to temperatures of 18, 25, and 29°C. By utilizing our recently developed approach[12], we identified thousands of previously unknown editing sites, many of which were temperature specific. Interestingly, we found that although editing is more prevalent at lower temperatures, more sites are edited at 29°C. This is due to loss of specificity of ADAR, as the sites edited at 29°C but not at lower temperature. At 29°C, the editing sites are less evolutionarily conserved, less frequently edited, less likely to be engaged in secondary structures and to target exons, and more likely to cause deleterious effects. Interestingly, dADAR hypomorphs cannot adapt their transcriptomes or behavioral patterns to temperature changes, indicating that editing is necessary for temperature adaptation. In sum, our work suggests a general role of ADAR as a sensor of secondary structure disruption, which is especially important during temperature adaptation.

## Results

### The degree and prevalence of A-to-I RNA editing is dynamically affected by temperature

**Fig 1:**
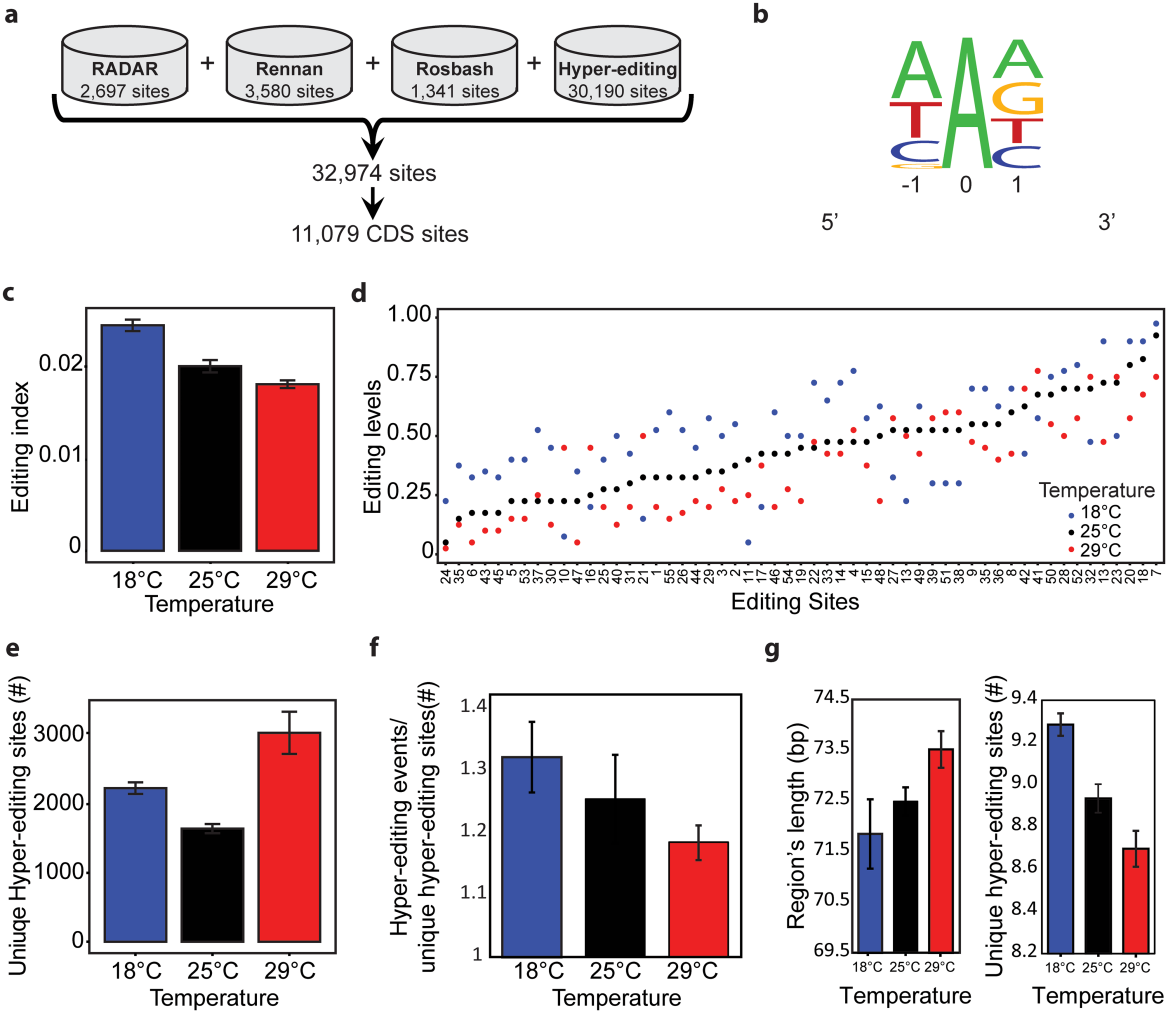
The degree and prevalence of A-to-I RNA editing are dynamically affected by temperature. **(A)** Generation of editing list by combining the RADAR database (2,697 sites), Rennan's and Rosbash's datasets[11,32] (3,580 and 1,341 sites respectively) with novel hyper-editing sites detected by our method (30,190 sites). This resulted in a list of 32,974 unique sites, containing 11,097 editing sites in CDS. **(B)** Hyper-editing motif. The sequence near the hyper-editing sites is depleted of Gs upstream and enriched with Gs downstream as expected from ADAR targets. **(C)** Editing index, fraction of inosines among all expressed adenosines of all detected editing sites, show lower editing levels at 29°C. **(D)** Editing levels of significantly altered 55 editing sites in CDS. Each site is presented by a number which indicates its position in S1 Table. **(E)** The distribution of hyper-editing detected sites, shows higher number of sites found at elevated temperature. **(F)** Average hyper-editing events per detected sites. Statistical significance between 18°C and 29°C was assessed by Student-t test (p<10^−4^). **(G)** Editing cluster's difference between temperatures. Left panel presents the average cluster length for each temperature. Right panel presents the average unique number of detected editing-sites for each temperature.

To explore the global effect of temperature on RNA-editing, we generated RNA-seq datasets from heads of flies entrained at three different temperatures (18, 25, and 29°C). To identify *de novo* RNA editing sites in this dataset, we utilized our previously published algorithm[12]. Our approach accurately detects edited and hyper-edited sequences without the need for matched DNA. Utilizing this approach, we detected 30,190 unique hyper-edited sites (Fig 1A). As expected, the A-to-G transitions were the most commonly observed and in all cases were localized in the expected strand of the DNA (see Methods, S1 Fig). The nearly 30,000 sites we identified far exceeds the 2,697[6], 3580[32], and 1,341[11] editing sites previously identified in flies. As expected, we were able to detect many of the previously identified editing sites (687 of 2,697 sites previously identified by RADAR[6], 1,181 of 3,580 sites and 332 of 1,341 sites in the other available datasets[11,32]), but the majority identified sites (83.95%) are novel, expanding the total number of known editing sites in fly to 32,974. Notably, more than a third of the newly identified sites (11,079) are in coding sequences (Fig 1A). This is a much higher percentage than any studied organism so far. Yet, the total number of recoding editing sites is lower than identified in Cephalopods [33]. Importantly, the sites exhibited a strong sequence consensus around the edited adenosine, similar to the previously described ADAR motif: a strong depletion of G immediately upstream to the edited site and some enrichment in G immediately downstream (Fig 1B).

When comparing editing events in flies entrained at different temperatures, we observed that the editing index (fraction of I/total number of reads) was lower at 29°C than at 18 or 25°C (Fig 1C), as previously described[32]. Importantly, we observed this trend both for all sites (32,974) as well as for only the newly identified hyper-edited sites (S2A and S2B Fig). Indeed, we detected 55 sites with significantly different editing efficiencies at different temperatures, a subset of which we also validated (Fig 1D and S3 Fig). Fifty of those 55 sites were also detected as edited in additional fly RNA-seq datasets (see Methods).

Surprisingly, and despite the lower editing index at 29°C compared to lower temperatures, there were significantly more hyper-edited sites at 29°C (3,036 sites) than at 25°C or 18°C (1,644 sites and 2,232 respectively; Fig 1E). These results show that although there is less hyper-editing activity overall, there are more hyper-editing sites at elevated temperature. As expected, we found that the average number of hyper-editing events per detected site at 29°C are significantly lower than at 18°C (p<10^−4^, Fig 1F).

The lower editing index and higher abundance of editing we observed at 29°C suggest that ADAR is more promiscuous at that temperature. As ADAR enzyme tends to edit sites in clusters[2], we explored whether the size of the clusters differed as a function of temperature. We defined cluster length as the number of nucleotides between the first and the last high-quality A-to-G mismatch (cluster should cover at least 10% of the read). Then, all overlapping hyper-edited clusters were merged to create a hyper-edited region, maintaining a maximum distance of 20 bases between clusters (S2D Fig). We found that the length of the hyper-edited region increased with temperature (Fig 1G left) and that the average number of edited sites within a cluster decreased as the temperature increased (Fig 1G right). In addition, there was a higher overlap between clusters at low temperature than at elevated temperature. Together, these results suggest that at high temperature the editing events are sparser and that ADAR is less specific.

### Editing sites at 29°C are less evolutionarily conserved and are not enriched in genes engaged in brain-specific function

**Fig 2:**
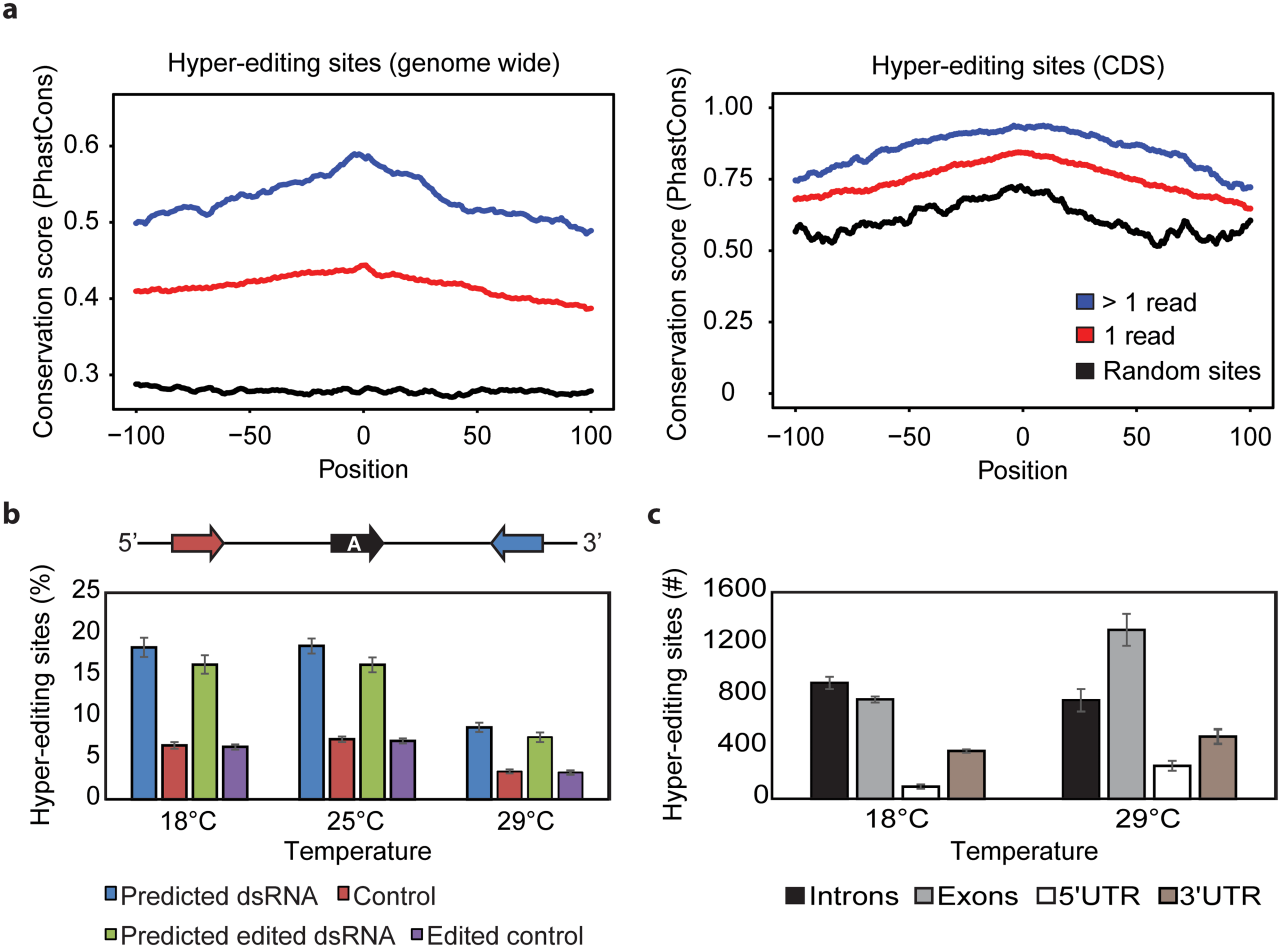
Editing sites at lower temperatures are edited more frequently and are more commonly flanked by complementary sequences. **(A)** Mean conservation (PhastCons) score of hyper-edited sites. Position 0 indicates the position of editing site. Blue line denotes conservation mean for editing sites supported by more than one event, red line denoted conservation mean for editing sites supported by only one event, and black line represents background conservation of chosen randomly adenosines. Left figure represents all genome wide hyper-editing sites, while the right figure represents hyper-editing sites in coding regions (CDS). The information from the non-hyper-edited reads was included. **(B)** RNA secondary structure prediction using BLAST[50] tool (see Methods). Blue bars donate for predicted dsRNA structure involving the hyper-editing site, as we succeeded to match the editing regions with their anti-sense sequence. Red bars denote for matches found in the sense sequence, representing the control. Green bars denote for predicted dsRNA structure involving the hyper-editing site after converting the adenosine (A) to its edited form, guanosine (G). Violet bars represents the control for the converted adenosines. **(C)** Genomic locations of detected hyper-editing sites show increase in the number of exonic sites at 29°C.

The presence of significantly more editing sites that are less frequently modified at 29°C than at lower temperatures raised the question of whether these sites might be modified in a stochastic way. Hence, we assessed the evolutionary conservation of the unique and higher frequency sites observed at 29°C. Indeed, conservation analysis (PhastCons[34] score) of the hyper-edited sites revealed that sites supported by more than one event were more likely to be evolutionarily conserved than unique hyper-edited sites (Fig 2A). This supports our hypothesis that uniquely edited sites, which are more abundant at higher temperature, are likely the results of non-specific activity of ADAR.

To identify whether transcripts from different functional classes are edited at different temperatures, we evaluated enrichment of gene ontology (GO) terms for mRNAs edited at the different temperatures using the PANTHER classification system[35]. We observed that edited transcripts at both 18 and 29°C are highly enriched for genes related to 'alternative splicing', 'nucleotide binding', and 'ATP binding' (Table 1). Interestingly, only the set of genes edited at 18°C is significantly enriched for transcripts with the 'RNA editing' term, given to transcripts with known edited sites (FDR < 0.05; Table 1). This again suggests that many of hyper-edited sites found in flies entrained at 29°C are random. As most of the characterized ADAR editing sites are in genes involved in neural function, it was not surprising that at 18°C we found a strong and significant enrichment for editing sites in genes related to 'ion transport', 'ion channels', and 'synapses' (Table 1). However, many of those terms are not significantly enriched in genes with transcripts edited at 29°C (Table 1), suggesting that at high temperature editing follows different rules or no rules.

**Table 1:** Functional analysis of hyper-edited genes.

Term	18°C p.value	18°C FDR	29°C p.value	29°C FDR
Alternative splicing	2.40E-12	3.20E-09	6.00E-16	7.30E-13
Phosphoprotein	7.20E-08	9.50E-05	1.70E-06	2.30E-03
Kinase	1.70E-07	2.20E-04	3.40E-07	4.50E-04
RNA editing	5.00E-07	6.50E-04	3.20E-04	N.S
Nucleotide-binding	4.40E-06	5.80E-03	1.40E-08	1.80E-05
Cytoplasm	5.40E-06	7.10E-03	7.20E-05	N.S
Ion transport	6.80E-06	8.90E-03	3.00E-02	N.S
Ionic channel	1.00E-05	1.30E-02	N.S	N.S
ATP-binding	1.50E-05	2.00E-02	5.80E-08	7.70E-05
Membrane	1.60E-05	2.00E-02	NA	NA
Cell junction	2.30E-05	3.00E-02	N.S	N.S
Synapse	2.40E-05	3.10E-02	N.S	N.S
Serine/threonine-protein kinase	5.40E-05	N.S	8.30E-08	1.10E-04
Nucleus	3.00E-04	N.S	1.30E-05	1.70E-02

Editing sites in both temperatures are present in mRNAs with specific functions. P.value and FDR are indicated for each functional category.

### Increase in temperature results in decreased ADAR specificity for inverted repeats

It is assumed that edited sites are located in double-stranded structures as ADAR binding and enzymatic activity is specific for these regions[28,36]. To determine whether the observed editing sites are located in regions of secondary structure, we determined whether there is a complementary RNA stretch on the same strand within 2 kilobases up- or downstream of the edited sites. For many of the edited sites, there is a nearby RNA sequence that could form a double-stranded structure that involves the edited site (Fig 2B, blue bars). As a control, we searched for complementary sequences in the opposite strand and found little complementarity (Fig 2B, red bars). This strongly suggests that edited sites must be involved in a double-stranded structure.

Interestingly, a higher percentage of editing sites are in potentially double-stranded regions at 18°C than at 29°C (18.41% and 8.73% of the hyper-edited sites, respectively). These data again suggest that although there are more editing sites at 29°C, ADAR is less specific at this temperature and may modify RNA even when it is not involved in a strong secondary structure. It is possible that at higher temperatures labile double-stranded RNA structures also serve as ADAR substrates. As expected based on previously results[32], we found that the *ADAR* mRNA expression decreased as temperature increased (S2C Fig), implying a potential mechanism for avoiding widespread non-specific editing at 29°C. One may argue that few editing sites have no dramatic impact on the dsRNA structure. However we noted that editing at even a single site has a detectable (although small) outcome on the predicted double strand structure (Fig 2B, green bars).

Although the results obtained above suggest the presence of little or no secondary structure on ADAR targets which are unique to 29°C, it is possible that secondary structure is present and was not detected by our aligning approach. Therefore, we determined the percentage of editing sites engaged in evolutionarily conserved secondary structures as predicted by EvoFold[37]. Consistent with the data presented above, we observed that at 18°C editing sites were twice as likely to be present in regions of evolutionarily conserved secondary structure than were sites edited at 29°C (Table 2).

**Table 2:** EvoFold analysis of hyper-editing sites.

Temperature	number of samples	Total number of sites	Number of evoFold sites	% evoFold sites
18°C	3	10,122	67	0.66%
25°C	2	5,406	27	0.50%
29°C	3	15,724	55	0.35%

Editing sites at low temperatures are more abundant inside evolutionary conserved functional RNA structures.

We next evaluated the regions of the pre-mRNA in which editing occurs at the different temperatures. Although the analyzed data were based on polyA^+^ selection, we observed a significant fraction of editing in intronic sequences. Indeed, we observed that at 18°C, 40% of the editing sites are located in intronic sequences. This is likely an underestimation, as we did not use nascent RNA for this assessment. Interestingly, at 29°C we observed a significant shift toward exons (Fig 2C). As exons tend to have fewer repeated sequences, and thus less option for stable long secondary structure than introns, these data again suggest more randomness in editing at 29°C. The obvious consequence of this miss-regulation is that of possible toxic effects of RNA editing at 29°C.

### ADAR hypomorphs display weaker adaptation of their transcriptome to temperature shifts

**Fig 3:**
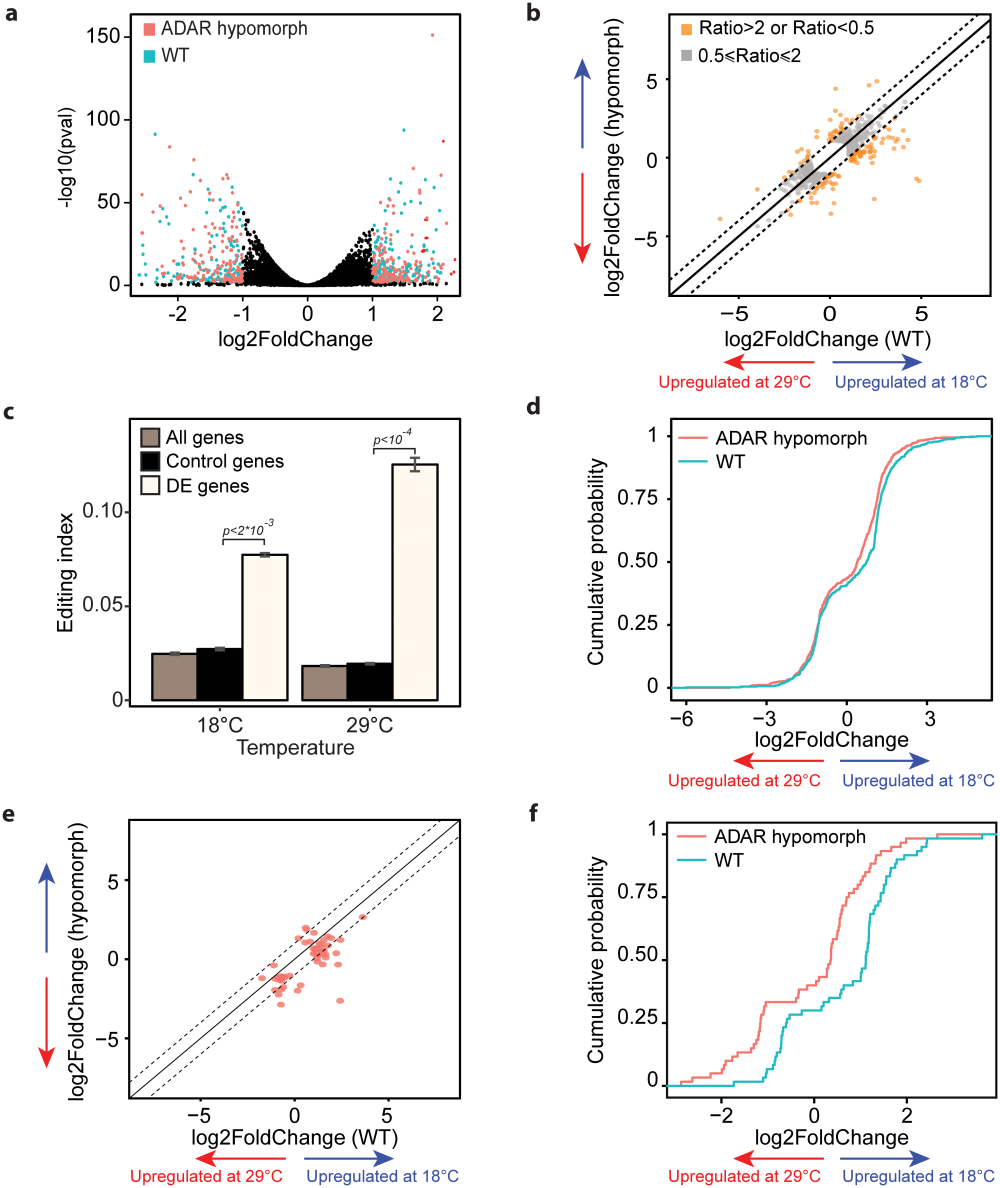
ADAR hypomorphs display weaker adaptation of their transcriptome to temperature shifts. **(A)** 'Volcano plot' showing gene expression differences in 29°C vs. 18°C in WT and ADAR hypomorph flies. Colored dots indicate genes significantly changing between the temperatures (p < 0.05, *log*_2_ (fold change) > 1). **(B)** Fold change levels of differentially expressed genes in 29°C vs. 18°C (selected as described in A) are plotted in a log scale WT against ADAR hypomorph. Orange dots indicate genes in which the fold change ratio between WT and ADAR hypomorph is > 2 or < 0.5 (**C**) Editing index, fraction of inosines among all expressed adenosines of all detected editing sites, for differentially expressed genes between control and ADAR hypomorph flies (light bars). Brown bars donate for editing index in all expressed genes. Black bars donates for editing index in control set of 60% most expressed genes. The p.values were calculated using bootstrapping exam with 10,000 random sampling from the control set. The editing levels were calculated on *wild type* CantonS strain. (**D**) Cumulative distribution plot for all genes differentially expressed between 29°C and 18°C. Kolmogorov-Smirnov test was used to determine the statistical significance of the differences between the curves (p < 0.0001). (**E**) Fold change levels of differentially expressed genes in 29°C vs. 18°C that are affected by ADAR hypomorph only in 18°C. Plot was generated as described in B. (**F**) Cumulative distribution plot of genes differentially expressed at 29°C vs. 18°C that are affected by ADAR hypomorph only in 18°C. Kolmogorov-Smirnov test was used to determine the statistical significance of the differences between the curves (p < 0.001).

Temperature provokes large changes in the fly transcriptome[38]. As ADAR activity and specificity are particularly affected by temperature, we sought to determine whether ADAR mediates part of the transcriptional response to temperature changes. We used ADAR hypomorph flies, which contain a transposon insertion in an *adar* intron and display a substantial reduction in ADAR levels and activity [39]. We analyzed gene expression in heads from control and ADAR hypomorph flies[39] entrained at 18°C and 29°C by 3’ RNA-seq. As expected, ADAR hypomorph flies displayed approximately 15% of the levels of editing observed in the control flies[39] (S4A Fig). This is likely due to lower RNA and protein levels and not the production of a new truncated protein, since we did not observe a new shorter *adar* mRNA isoforms in the hypomorph flies, but we detected significantly lower levels of *adar* mRNA (S4B and S4C Fig). Next, we examined the differential gene expression between 29°C and 18°C in WT vs. ADAR hypomorphs. As previously described[38], we observed significant differences in gene expression between the two temperatures (Fig 3A), and there was a good correlation in the changes of mRNA expression in the two strains (Fig 3B). To determine whether differences in RNA editing could lead to specific changes in the fly transcriptome we determined the gene editing levels of the genes differentially expressed between control and ADAR hypomorph flies. Indeed, we found that the differentially expressed genes exhibited significantly higher levels of editing than the control set of genes (most expressed genes, Fig 3C). Interestingly, that behavior was also influenced by temperature, as editing levels in those differentially expressed genes were significantly higher at 29°C (p = 2*10^−3^, Fig 3C).

While performing the differentially expression analysis we observed a group of genes that responded differently to temperature changes (Fig 3B, orange dots). More specifically, we observed significant differences in the mRNAs that are upregulated at 18°C in wild-type flies, and found that the temperature dependent changes are of smaller magnitudes in the dADAR hypomorph flies (Fig 3D).

In order to detect the source of this variation we divided the temperature affected genes into groups based on their expression levels in the dADAR hypomorph flies comparing to WT. Obviously, genes with similar expression levels in WT and dADAR hypomorph flies at both 18°C and 29°C will have also similar fold change values when comparing between temperatures in each strain. This means that the bias we observed in the fold change levels as described in Fig 3D must be related to genes differentially expressed in dADAR hypomorph in at least one of the temperatures. Interestengly, we found that this bias is related to the group of genes that are differentially expressed in dADAR hypomorphs only in 18°C (Fig 3E and 3F), but not to the ones differentially expressed in 29°C or in both temperatures (S4D and S4E Fig). Since ADAR expression levels are higher in 18°C this may imply that this group of genes requires higher ADAR levels. These results suggest that ADAR, directly or indirectly, mediates or amplifies an important part of the transcriptional response induced by temperature changes.

### ADAR modulates the behavioral adaptation to temperature changes

Locomotor activity is strongly influenced by temperature. *Drosophila melanogaster* flies are mainly active during the day at 18°C but quickly become nocturnal after exposure to 29°C[29]. To determine whether ADAR mediates this response we measured the locomotor activity patterns of control and ADAR hypomorph flies at 18°C and 29°C.

**Fig 4:**
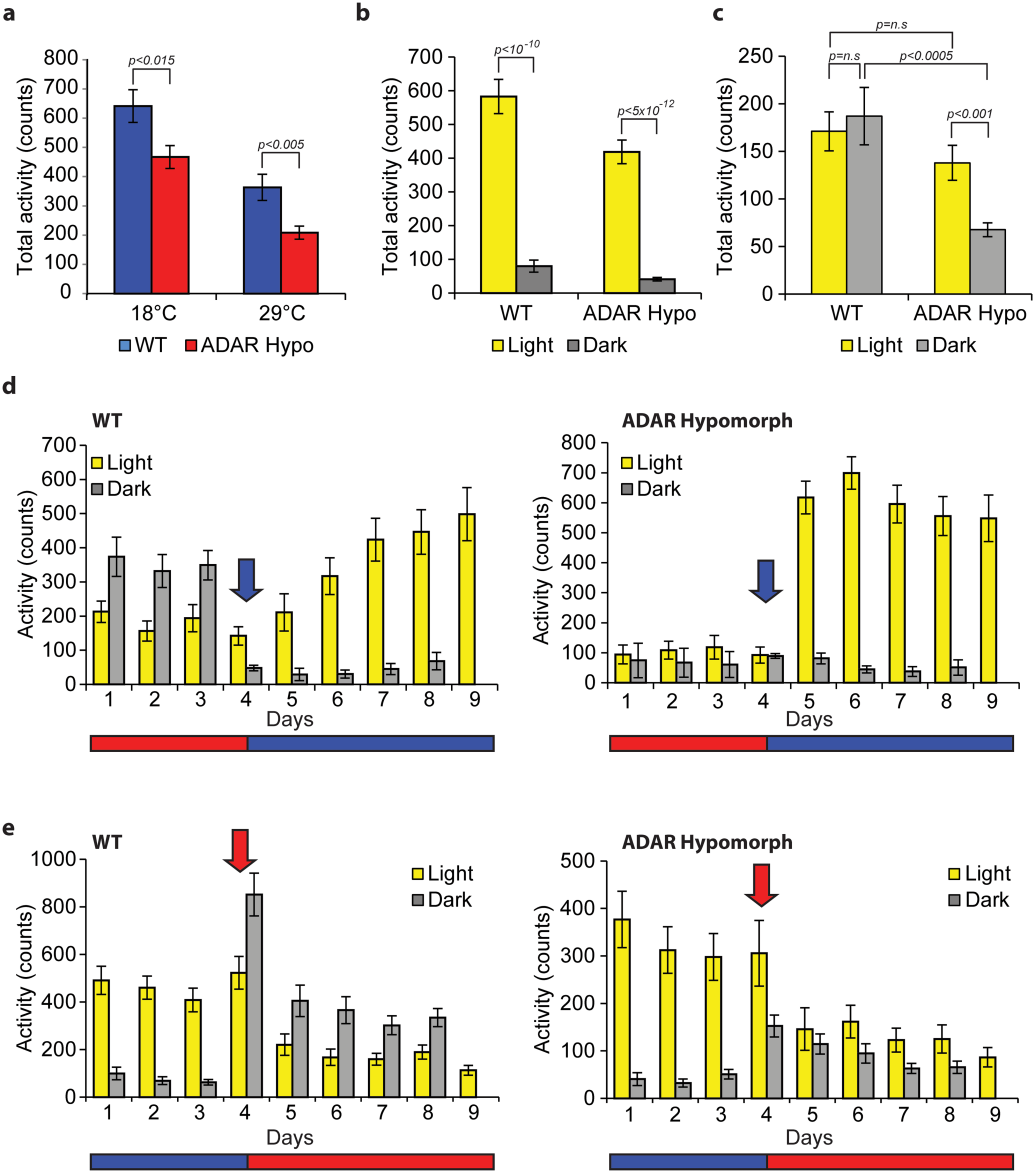
ADAR hypomorph flies display temperature dependent behavioral abnormalities. **(A)** ADAR hypomorph flies (red) are less active than control flies (blue) both at 18°C and 29°C. Total activity per day obtained by adding the average activity during the light and dark periods (8 days). N = 32 and 29 for hypomorph flies at 18°C and 29°C respectively and N = 27 for control flies at both temperatures. Statistical significance was assessed by Student-t test. Error bars represents SEM. **(B)** Although less active than their controls, at 18°C, the pattern of day-night activity of ADAR hypomorph and control flies is similar, with higher activity during the day. We calculated and ploted the average activity during the light (9 days) or dark periods (8 nights). Statistical significance was assessed by Student-t test. Error bars represents SEM. **(C)** At 29°C, control flies increase their night activity whereas the ADAR hypomorph flies remaine active mostly during the day. Statistical significance was assessed by Student-t test. Error bars represents SEM. **(D)** Behavioral activity assay for control (left) and ADAR hypomorph flies (right) that were exposed to 12:12h light:dark (L:D) cycles at 29°C for 4 days and then transferred to 18°C (L:D cycles) for 5 days. N = 29 for control and N = 32 for Adar hypomorph flies. An arrow marks the transition time point. Error bars represent SEM. **(E)** same as in (D), with the opposite temperature transfer, from 18 to 29°C. N = 30 for control and N = 31 for ADAR hypomorph flies. An arrow marks the transition time point.

We found that ADAR hypomorph flies were less active than control flies both at 18°C and 29°C (Fig 4A). This was expected based on the previously described defects in locomotor activity of the ADAR hypomorph flies at 25°C[39]. At 18°C ADAR hypomorph flies displayed a pattern of activity similar to that of the control flies with most activity during the light period (Fig 4B). At 29°C, control flies were equally active during the dark and lights-on periods, whereas the ADAR hypomorph flies remained active mostly during the day, demonstrating that this strain fails to completely adapt to higher temperature (Fig 4C).

To determine the kinetics of the adaptation to temperature, we performed additional experiments in which we recorded the behavioral patterns of control and ADAR hypo flies before and after transferring from 29°C to 18°C or vice versa. The control flies adapted to 18°C by increasing the day-time activity monotonically over the first few days at this temperature (Fig 4D, left, and S5 Fig). In contrast, ADAR hypomorph flies increased day-time activity immediately after transfer (Fig 4D, right, and S5 Fig), suggesting that ADAR activity somehow smoothens the behavioral transition to 18°C. In addition, when flies were transferred from 18°C to 29°C, the response of the ADAR hypomorphs was severely diminished (Fig 4E), suggesting an important role of ADAR in regulating the overall adaptation to higher temperatures. Interestingly, these differences were only detected at the activity level. We did not observe significant differences in the total amount of sleep during the day or night between ADAR hypomorphs and the control strain (S5 Fig).

## Discussion

Here we demonstrate a role for A-to-I editing and specifically the enzyme ADAR in temperature adaptation in *Drosophila*. We showed that temperature not only changes the total levels of editing but also the specificity of this modification. Briefly, we found that despite a higher level of editing at lower temperatures, more individual sites were edited at 29°C than at 18 or 25°C. This is due to a lower level of ADAR activity at 29°C. The sites modified at the high temperature were less evolutionarily conserved, more dispersed, less likely to occur in regions of secondary structure, and more likely to be located in exons. These results strongly support the notion that at 29°C, RNA editing is less deterministic and might even have deleterious effects.

Interestingly, hypomorph mutants for ADAR display a weaker transcriptional response to temperature changes than wild-type flies. In addition, and in agreement with the differences observed in the head transcriptomes, ADAR hypomorph flies displayed a highly abnormal behavioral response to temperature changes. In sum, our data show that ADAR is essential for proper temperature adaptation, a key behavioral trait that is essential for the survival of flies in the wild.

Recent work has shown that A-to-I editing is regulated by temperature with the higher levels detected at lower temperatures (15°C compared to 35°C)[30]. However, those studies were based on the study of only a handful of edited sites. Here we show that A-to-I RNA editing in *Drosophila* is much more common than previously proposed, and we provide the largest set of editing sites identified in this organism so far. Previous reports demonstrated that ADAR is negatively regulated at higher temperatures[30]. The decrease in A-to-I editing is due to lower dADAR protein levels as well as activity. Interestingly, the latter is achieved by temperature-sensitive auto-editing events[32]. These regulatory events cannot explain the loss in specificity of dADAR we observed at 29°C. It is possible that changes in ADAR activity induced by temperature mediate part of this loss of specificity. However we believe that these differences are rather due to changes in the secondary structure of the substrate RNA, which is generally altered by temperature *in vivo* [40,41]. The double-stranded structures that are dADAR substrates are likely more common at 18°C than at higher temperatures. Indeed, we found that a large fraction of the editing sites at 18°C are in regions predicted to be involved in secondary structure. We propose that ADAR has a role in regulating RNA secondary structure, likely during transcription but probably also later in the RNA life cycle. In mammals, A-to-I editing inhibits the formation of cytoplasmic RNA duplexes that could be interpreted by the cell as a sign of a viral infection[27].

The presence of strong secondary structures during transcription would have strong consequences both on transcription and other co-transcriptional events like splicing. As A-to-I editing happens co-transcriptionally, the possible disruption of specific RNA structures involving intronic sequences can influence splicing as well as polyadenylation efficiencies. Others and us recently showed that circular RNA (circRNA) production is regulated by ADAR in mouse[42] (ADD); dADAR likely regulates circRNA production in flies as well. Although circRNA production in fly heads is significantly increased at 29°C[43], we failed to find any correlation between circRNA levels and RNA editing in response to temperature changes (S2 Table). This does not rule out a role of dADAR in regulating circRNA levels, but rather suggests that the regulation is complex. A similar interplay might occur with alternative splicing and RNA editing or other types of mRNA processing events. Indeed, our finding that a subset of genes that are upregulated at 18°C (relative to 29°C) in wild-type flies were not expressed at significantly different levels in the ADAR hypomorph upon temperature shift, provides support for this hypothesis. It will be interesting to determine whether the splicing efficiency of these transcripts is influenced by temperature-dependent editing events.

The arguments laid out above might explain the necessity for high dADAR activity at 18°C. As putative disruptive structures in RNA transcripts must be quickly removed in order not to affect mRNA production, ADAR has evolved to be highly efficient at 18°C. The specificity of ADAR becomes problematic then at high temperatures as secondary structures are more labile. In this situation, ADAR activity increases the chance of mutations in exonic sequences, which we indeed found are more heavily edited at 29°C compared to 18°C. It seems plausible that ADAR is not completely necessary at 29°C; however, the behavioral defects observed in the ADAR hypomorphs suggest a more complicated mechanism. First, these mutant flies are generally less active than wild-type flies as previously described, and their activity levels are not temperature dependent. Second, after three days of entrainment ADAR hypomorphs adapt well to 18°C but not to 29°C. The lack of effect at 18°C might be related to the fact that the most important structures to be disrupted (and re-coding sites to be edited) likely engage in strong secondary structures at this temperature and that the remaining ADAR activity might suffice to disrupt them. The lack of adaptation to the higher temperature could be explained either by the presence of temperature-dependent re-coding events of important behavior-related genes or to the lack of disruption of structures that form at this temperature.

Previous studies have addressed roles for dADAR in fly behavior. While ADAR5G1 have completely disrupted locomotor behavioral patterns, they are likely of developmental origin, as dADAR hypomorph flies display more modest behavioral abnormalities[39,44,45]. dADAR hypomorphs also display abnormal male courtship behavior[39] and sleep[46]. These reported behavioral defects are likely due to missediting of transcripts encoding key proteins as the ones encoded by the genes *fruitless*[39], *cacophony*[44] or unknown mRNAs present in glutamatergic neurons[46].

In our observation of the kinetics of the adaptive response to temperature changes, another role for dADAR becomes apparent. In control flies, the temperature change *per se* resulted in an immediate behavioral response (decrease or increase in night activity for flies being transfered from 29°C to 18°C or from 18°C to 29°C, respectively; see S5 Fig). These immediate responses are much attenuated in the ADAR hypomorph flies (even when the overall response is increased in some of the cases; see S5 Fig). This is particularly evident in the 29 to 18°C transition and suggests that disruption of specific secondary structures by ADAR might be part of the temperature-sensing mechanism or at least involved in the early response to temperature changes. Interestingly, after this abrupt inhibition of the night activity, wild-type flies steadily increased the daily activity on the following days, whereas ADAR hypomorphs achieved the full response in the first day following the temperature transition. In the transition from 18 to 29°C, despite the abnormal behavioral response of ADAR hypomorphs, the kinetics of the transition were similar in both flies with both strains fully displaying their pattern of activity within the first day. We believe that the difference in adaptation of the flies to 29°C might be related to the lack of re-coding of specific mRNAs. However, we believe that the transition to lower temperatures requires a more general role of ADAR in maintaining the folding homeostasis of the RNA. The fact that ADAR hypomorph flies retain some of the molecular and behavioral responses to temperature strongly suggests that the observed effects are not affecting the general pathways for sensing temperature.

## Materials and methods

### Fly stocks

For the identification of temperature dependent editing events, we utilized a *wild type* CantonS strain (Bloomington stock center, Indiana). We also utilized a *wt* and ADAR hypomorph strains[39] (*Adar*^*5G1*^).

### RNA extraction and RNA-libraries preparation

Total RNA was extracted using Trizol reagent (Sigma) and treated with DNase I (NEB) following the manufacturer's protocol.

Stranded ligation-based, total-RNA libraries preparation was modified from[47] as follows: 1μg of total RNA was polyA^+^ selected (using Oligo(dT) beads, Invitrogen), fragmented in FastAP buffer (Thermo Scientific) for 3min at 94°C and then dephosphorylated with FastAP, cleaned (using 2.5X volume on SPRI beads, Agencourt) and then ligated to a linker1 (5Phos/AXXXXXXXXAGATCGGAAGAGCGTCGTGTAG/3ddC/, XXXXXXXX is an internal barcode specific for each sample), using T4 RNA ligase I (NEB). Ligated RNA was cleaned-up by Silane beads (Dynabeads MyOne, Life Technologies) and pooled into a single tube. RT was then performed for the pooled sample, with a specific primer (5´-CCTACACGACGCTCTTCC-3´) using AffinityScript Multiple Temperature cDNA Synthesis Kit (Agilent Technologies). Then, RNA-DNA hybrids were degraded by incubating the RT mixture with 10% 1M NaOH (e.g. 2ul to 20ul of RT mixture) at 70C for 12 minutes. pH was then normalized by addition of corresponding amount of 0.5M AcOH (e.g. 4ul for 22 ul of NaOH+RT mixture). The reaction mixture was cleaned up using Silane beads and second lygation was performed, where 3’end of cDNA was ligated to linker2 (5Phos/AGATCGGAAGAGCACACGTCTG/3ddC/) using T4 RNA ligase I. The sequences of linker1 and linker2 are partially complementary to the standard Illumina read1 and read2/barcode adapters, respectively. Reaction Mixture was cleaned up (Silane beads) and PCR enrichment was set up using enrichment primers 1 and 2 (5’-AATGATACGGCGACCACCGAGATCTACACTCTTTCCCTACACGACGCTCTTCCGATCT-3’,

5’-CAAGCAGAAGACGGCATACGAGATXXXXXXXXGTGACTGGAGTTCAG ACGTGTGCTCTTCCGATCT-3’, where XXXXXXX is barcode sequence) and Phusion HF MasterMix (NEB). 10–12 cycles of enrichment were performed. Libraries were cleaned with 0.7X volume of SPRI beads. Libraries were characterization by Tapestation. RNA was sequenced as paired-end samples, in a NextSeq 500 sequencer (Illumina).

**For 3’ Digital gene expression** libraries preparation was similar, with one exception- PolyA^+^ selection was not done before fragmentation, but after linker1 ligation and samples pooling (before the RT reaction step).

### Editing validation

RNA (DNaseI treated, NEB) extracted from flies entrained at 18°C/ 25°C/ 29°C was reverse transcribed using random primes (iScript cDNA Synthesis kit, Bio-Rad). The resulting cDNA, and genomic DNA from the above flies were amplified by PCR (KAPA HiFi, KAPAbiosystems) using the following primers:

Sh-RO (intronic): Fwd: 5’-CTCCGGACCCCAAATCTAAC-3’, Rev: 5’-CGTTTCGCGGTGATAGAAGT-3’;

Calx-RF: Fwd: 5’-GGACAAGAACTACCGGGTCA-3’, 5’-AGTACATTCGGATGGGATCG-3’;

Ca-beta: Fwd: 5'-ACTCCCAGTCCCACTCTCAGTAT-3', Rev: 5'- GTGGATGTATCTGTGTCGCTGTA -3';

Tut1: Fwd: 5'- TTAGTATCGCACGAATCGGTATC -3', Rev: 5'- GACCTACTACTTCCGCGTGCT -3';

nAChRa1pha6: Fwd: 5'- ATAACCGATGAATCGAACTGATG -3', Rev: 5'- TTTGCTGTGTATTTTGTCGTTTG -3';

NaCP60E: Fwd: 5'- AATATTCCTTCCAGCCCGTTT -3', Rev: 5'- CCATCTGATTTACTTGCAGATACG -3';

Unc80: Fwd: 5'- GGCGGATTCTGAGACATGAG -3', Rev: 5'- CTGCTAAAGGTGTCGCCCTAT -3';

CG8481: Fwd: 5'- GAGGCAATCACACATGCACTT -3', Rev: 5'- ATATATCTGTCCACCATCGACCA -3';

Para: Fwd: 5'- TACCAAACTCCAAACCCCTTATT -3', Rev: 5'- TCTTTGTATAACGCTCACCGACT -3';

DopEcR: Fwd: 5'- GAGAACAACATGACGCACATTT -3', Rev: 5'- ATGATCAGAATTTTCCAAAACGA -3';

Amplified PCR products were cleaned from agarose gels and subjected to sanger sequencing.

### Read alignment

All reads at each file were aligned to dm3 genome, using BWA aln (0.6.2) with default parameters, and then BWA mem (0.7.4) with minimum seed length of 50. We kept only uniquely aligned reads (using samtools). The unaligned reads were used for detection of hyper-editing events, and the aligned reads were used for further analysis.

### Hyper-editing analysis

We used the recently published pipeline[12] for detection of hyper-edited reads in RNA-seq data. This algorithm is specialized to identify editing clusters that are ignored by standard alignment tools. In the sequencing reaction we expect the reads to be equally distributed from both orientations and the hyper-editing events to have no position preference along the read. Indeed, unlike other types of mismatches, A-to-G transitions were equally distributed in both plus and minus strand. In addition, we see no position preference on the reads for A-to-G, whereas A-to-C transitions (background mismatch) tended to localize in specific position, suggesting sequence errors as a source for this bias (S1 Fig). Therefore, non A-to-G mismatches were eliminated in this work.

### Analyses of RNA editing levels

The analyses of editing levels were done using REDItools script[48], which systematically identifies RNA editing events from a given list of sites. To reduce sequencing errors, six bases were trimmed out up and down the reads. We demanded that at least 2 reads support A-to-I variation at an editing frequency of at least 1%. We generated an editing list by combining the RADAR database[6] (2,697 sites), Rennan's and Rosbash's datasets[11,32] (3,580 and 1,341 sites respectively) with novel hyper-editing sites detected by our method (30,190 sites). This resulted in a list of 32,974 unique sites.

To detect RNA-editing sites in coding regions, we annotated all sites using ANNOVAR[49] and then excluded those sites in non-coding sequences. This yielded 11,079 sites. Statistical significance of alteration between temperatures for each editing event was evaluated using the *χ*^2^ test followed by 5% FDR correction. Then we compiled a list of 55 significantly altered editing sites in coding sequence, with at least 50 reads coverage and over 20% editing difference between temperatures.

Further validation on those sites was done using fly RNA-seq datasets downloaded from NCBI Gene Expression Omnibus database (accession number—GSE37232).

### Defining double-stranded RNA structures

In order to detect formation of double-stranded RNA structures, we searched for alignment of 40 bases centered on the hyper-edited sites with sequence 2 kilobases upstream and downstream of the regions. We used bl2seq[50] with -G -2 -F F -r 2 -W 7 -e 1000 parameters. A match was considered with 70% identity along 70% of the hyper-edited region length. To test the accuracy of this detection we calculated the ratio between matches of the hyper-edited regions and their antisense sequence (average of 15.24%) compared with matches to the same strand (average of 5.74%).

### Calculating expression levels

The number of aligned reads to each gene was calculated using featureCounts[51], with the same alignment. The expression level of dADAR was calculated using DEseq package[52] in R.

### Differential gene expression analysis from 3’DGE experiments

RNA-seq reads from 3’ Digital gene expression (DGE) experiments were aligned to the genome and transcriptome (dm3) using TopHat[53]. ESAT tool[54] was used in order to count number of reads for each gene, and differential expression analysis was performed with DEseq[52]. We considered genes with log(fold change)>1 and p-value<0.05 as significantly changing.

### Locomotor activity measurements

Male flies were monitored using Trikinetics *Drosophila* Activity Monitors using 1 minute bins. Flies were entrained 5 days in 12:12 Light:Dark cycles (LD). Before temperature shift (to 18 or 29°C), flies were entrained for 3 days at 25°C. Analyses were performed with a signal processing toolbox[55]. All the activity assessments were done in LD. Total activity for four days during the light/ dark phase was averaged

### Sleep

Adult male flies (3–5 days old) were placed in glass tube and monitored for 5–6 days in constant light-dark (LD) condition via using Trikinetics *Drosophila* Activity Monitors (DAM; Trikinetics,Waltham,MA) system. During the experiment, flies were supplemented with agar food (2% Agar and 5% sucrose). The sleep assay was performed at fixed humidity and temperature (25 ± 1°C). Data were collected according to pySolo manual and analyzed via pySolo sleep analysis software[56].

### Accession numbers

All raw sequencing data are available for download from National Center for Biotechnology Information GEO database under accession number GSE95313.

## Supporting information

S1 Fig Quality control of Data. **(A)** Count of hyper-editing events in *drosophila melanogaster*. Most of the detected mismatches were of A-to-G type. A total of 30,190 unique A-to-G editing sites were discovered, more than 74% of all the detected unique sites. 10,300 (25%) of the detected sites were of A-to-C type substitution, next was proven to be due to a sequencing error. **(B)** Distribution of hyper-editing events along read's positions. Each bar represents the number of editing events (A-to-G or A-to-C) found at every specific position within the read. We expect that real editing events to have no position preference along the read, whereas A-to-C substitution (background mismatch) tended to localize in specific position. Importantly, the distribution of A-to-G hyper-editing sites is not equal along the read since we discarded clusters too close to the end of the read to overcome improper alignment due to splicing [12]. **(C)** Distribution of editing events across the reads strands. Each bar refers to a specific type of mismatch, and shows the fraction of editing sites in each strand (+ or -) as well as the specified mismatch (e.g., A-to-G). Only the A-to-G sites show the expected behavior from true editing sites (~50/50%), while A-to-C substitution was detected only in the sense (+) strand, strongly suggesting that these mismatches are the result of technical error during the sequencing reaction.
(TIF)

S2 Fig Analysis of known and hyper-editing sites. **(A)** Venn diagram describing the number of detected hyper-editing sites in each temperature. 10,122 unique A-to-G editing events were discovered at 18°C, 5,406 at 25°C and 15,724 at 29°C. 681 (2%) of the detected hyper-editing sites were shared between all three temperatures. **(B)** The fraction of inosines among all expressed adenosines of previously detected hyper-editing sites, shows a lower editing levels at 29°C. **(C)** dADAR deferential expression levels distributions at different temperatures is shown. ADAR transcript levels decrease as temperature increases. **(D)** Illustration of hyper-edited region. Hyper-edited cluster defined as the number of nucleotides between the first and the last high-quality A-to-G mismatch (cluster should cover at least 10% of the read). All overlapping hyper-edited clusters were merged (the genomic coordinates) to create a hyper-edited region, maintaining a maximum distance of 20 bases between edited clusters. Region's boundaries were set from the first base of the upstream cluster, to the last base of the downstream cluster.
(TIF)

S3 Fig Editing alteration in coding sequences. **(A)** Each row represents one of the 55 editing sites in CDS (S1 Table) that were significantly modified between temperatures, and each column represents one tested sample. The editing levels are represented by the color in each position. The samples were clustered showing clear division between temperatures. **(B)** Validation of Calx (chr3R:16819460, CDS) and Sh (chrX: 17824987, intron) differential editing using direct Sanger sequencing. **(C)** Genomic validations of eight significantly altered editing sites: Ca-beta (chr2L:11159274), tut1 (chr2L:4314586), nAChRalpha6 (chr2L:9809350), NaCP60E (chr2R:20802317), unc80 (chr3R:23500290), CG8481 (chr3R:5595147), para (chrX:16398273), DopEcR (chr3L:4369459).
(TIF)

S4 Fig *Adar* is required for adaptation of the transcriptome to temperature changes. **(A)** The distribution of hyper-editing detected sites, normalized to the aligned number of reads, shows a different editing behavior for hypomorfic and WT flies. **(B)** ADAR hypomorph flies have lower expression of *Adar* mRNA comparing to WT flies. The data presents normalized read counts from 3' RNA-seq experiment **(C)** IGV snapshot of RNA-seq data from WT and ADAR hypomorph flies 3'. The single peak in both strains demonstrates no production of a new truncated protein in the ADAR hypomorph flies **(D)** Cumulative distribution plot for genes differentially expressed in 29°C vs. 18°C that are affected by ADAR hypomorph in both 18°C and 29°C. **(E)** Cumulative distribution plot for genes that are affected by ADAR hypomorph only in 29°C. For the comparisons presented in B and C Kolmogorov-Smirnov test showed that there are no significant differences between the curves (p = 0.83 and p = 0.69, respectively).
(TIF)

S5 Fig ADAR hypomorph flies show no differences in their sleep in respect to their controls. **(A)** No difference in average sleep between ADAR hypomorph and control flies, both at 18 and 29°C. **(B)** No difference in night or day sleep between ADAR hypomorph and control WT flies at 18 or 29°C. **(C-D)** Temperature transition (marked in an arrow) does not affect total sleep of ADAR hypomorph flies, in respect to their controls.
(TIF)

S1 Table CDS editing analysis. (XLSX)

S2 Table RNA editing inside circRNA. (XLSX)
