# Supplementary figures and images for: Dynamic hyper-editing underlies temperature adaptation in Drosophila
Source: PLoS Genet. 2017 Jul 26;13(7):e1006931. doi: 10.1371/journal.pgen.1006931 (PMC5550009; doi:10.1371/journal.pgen.1006931)

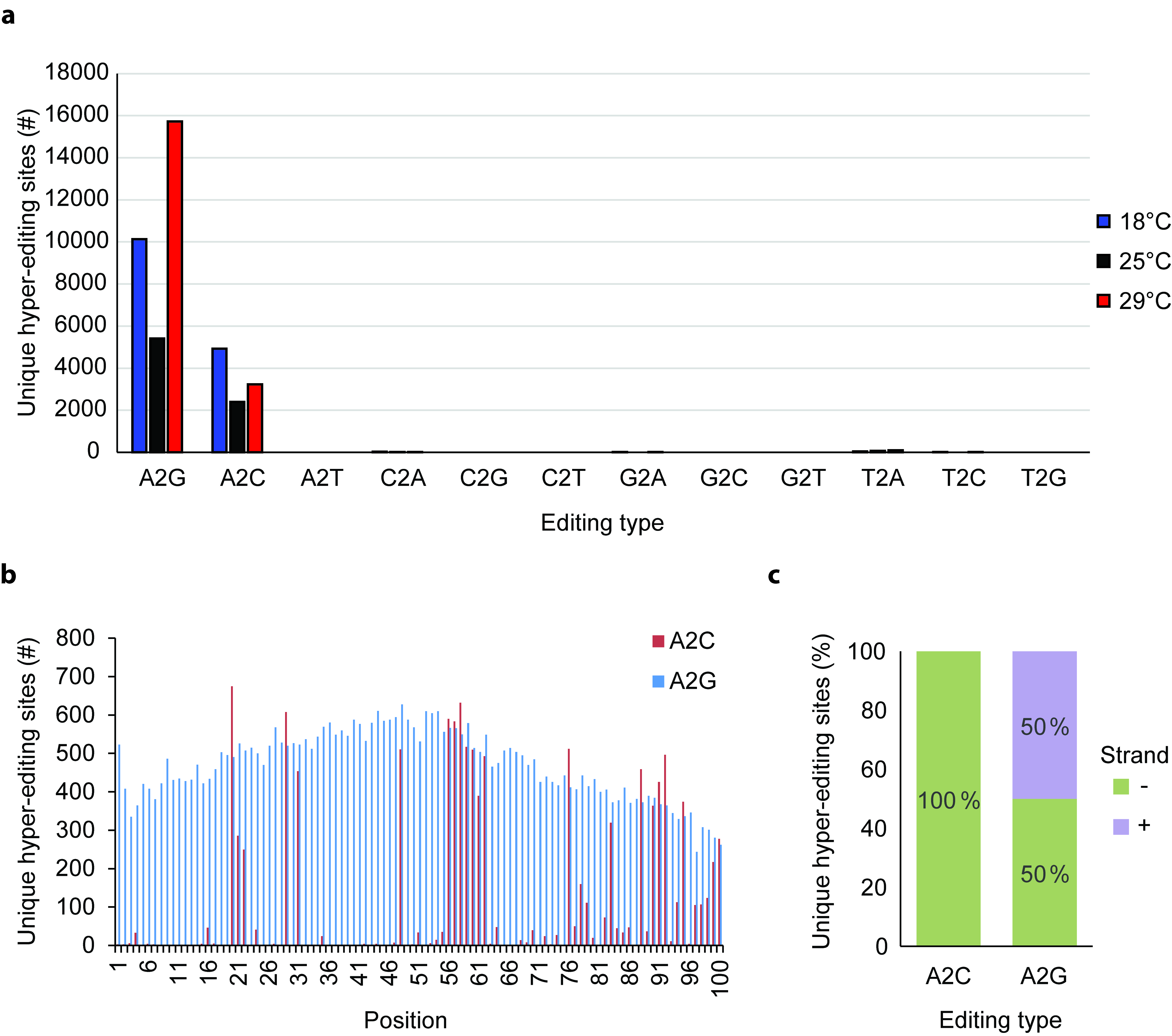

Supplement: S1 Fig — (A) Count of hyper-editing events in drosophila melanogaster. Most of the detected mismatches were of A-to-G type. A total of 30,190 unique A-to-G editing sites were discovered, more than 74% of all the detected unique sites. 10,300 (25%) of the detected sites were of A-to-C type substitution, next was proven to be due to a sequencing error. (B) Distribution of hyper-editing events along read's positions. Each bar represents the number of editing events (A-to-G or A-to-C) found at every specific position within the read. We expect that real editing events to have no position preference along the read, whereas A-to-C substitution (background mismatch) tended to localize in specific position. Importantly, the distribution of A-to-G hyper-editing sites is not equal along the read since we discarded clusters too close to the end of the read to overcome improper alignment due to splicing [12]. (C) Distribution of editing events across the reads strands. Each bar refers to a specific type of mismatch, and shows the fraction of editing sites in each strand (+ or -) as well as the specified mismatch (e.g., A-to-G). Only the A-to-G sites show the expected behavior from true editing sites (~50/50%), while A-to-C substitution was detected only in the sense (+) strand, strongly suggesting that these mismatches are the result of technical error during the sequencing reaction. (TIF) [file pgen.1006931.s001.tif]

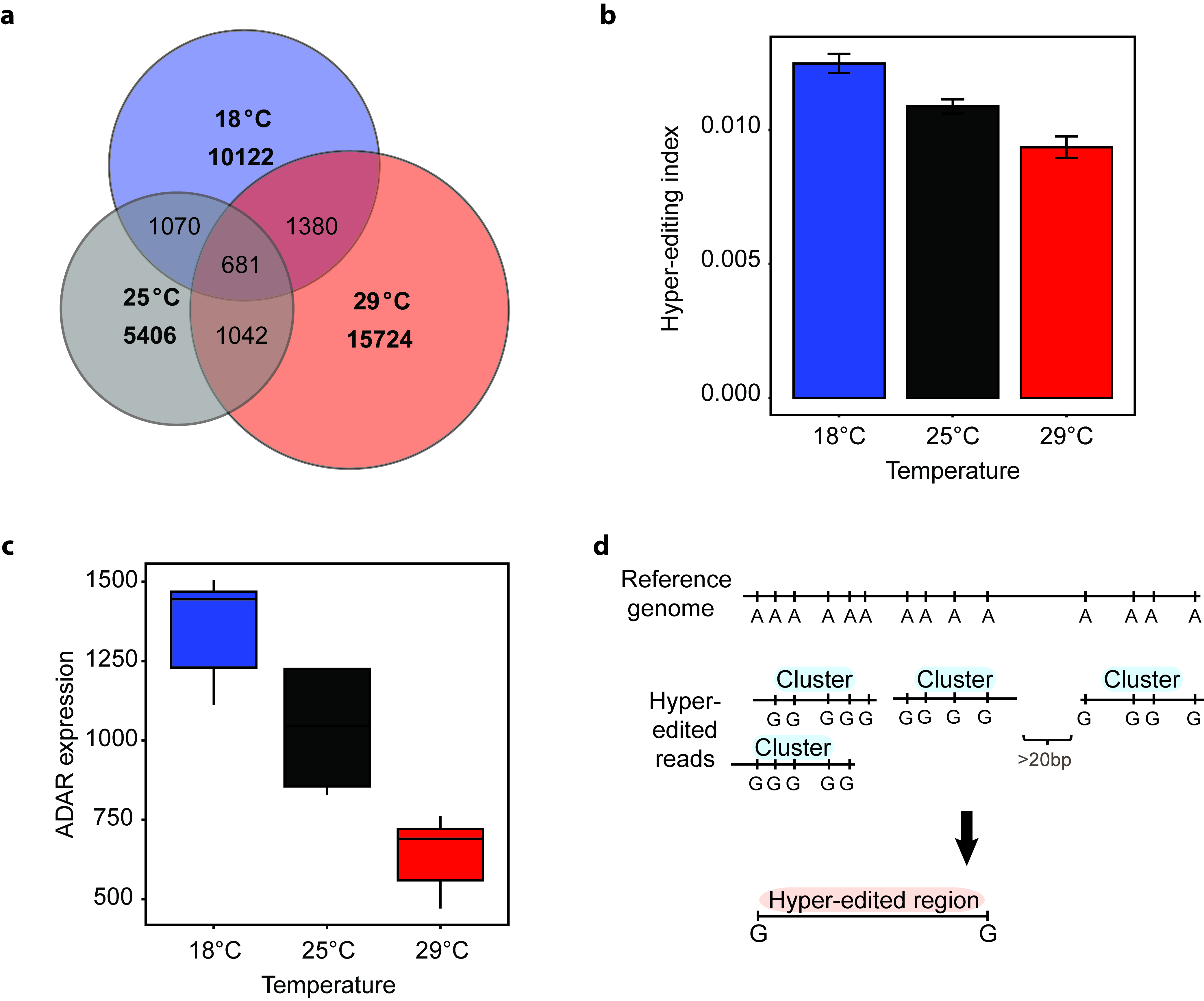

Supplement: S2 Fig — (A) Venn diagram describing the number of detected hyper-editing sites in each temperature. 10,122 unique A-to-G editing events were discovered at 18°C, 5,406 at 25°C and 15,724 at 29°C. 681 (2%) of the detected hyper-editing sites were shared between all three temperatures. (B) The fraction of inosines among all expressed adenosines of previously detected hyper-editing sites, shows a lower editing levels at 29°C. (C) dADAR deferential expression levels distributions at different temperatures is shown. ADAR transcript levels decrease as temperature increases. (D) Illustration of hyper-edited region. Hyper-edited cluster defined as the number of nucleotides between the first and the last high-quality A-to-G mismatch (cluster should cover at least 10% of the read). All overlapping hyper-edited clusters were merged (the genomic coordinates) to create a hyper-edited region, maintaining a maximum distance of 20 bases between edited clusters. Region's boundaries were set from the first base of the upstream cluster, to the last base of the downstream cluster. (TIF) [file pgen.1006931.s002.tif]

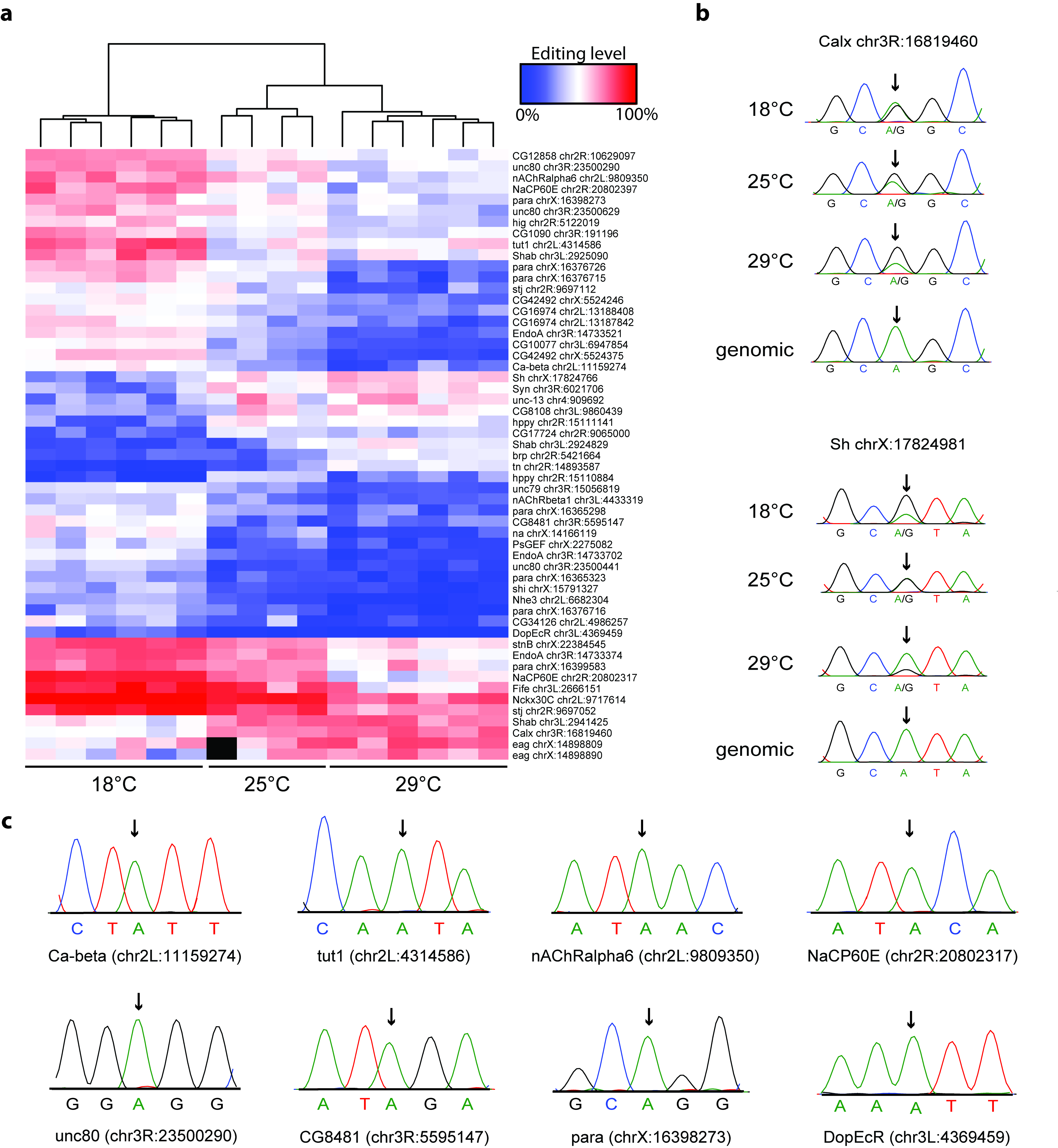

Supplement: S3 Fig — (A) Each row represents one of the 55 editing sites in CDS (S1 Table) that were significantly modified between temperatures, and each column represents one tested sample. The editing levels are represented by the color in each position. The samples were clustered showing clear division between temperatures. (B) Validation of Calx (chr3R:16819460, CDS) and Sh (chrX: 17824987, intron) differential editing using direct Sanger sequencing. (C) Genomic validations of eight significantly altered editing sites: Ca-beta (chr2L:11159274), tut1 (chr2L:4314586), nAChRalpha6 (chr2L:9809350), NaCP60E (chr2R:20802317), unc80 (chr3R:23500290), CG8481 (chr3R:5595147), para (chrX:16398273), DopEcR (chr3L:4369459). (TIF) [file pgen.1006931.s003.tif]

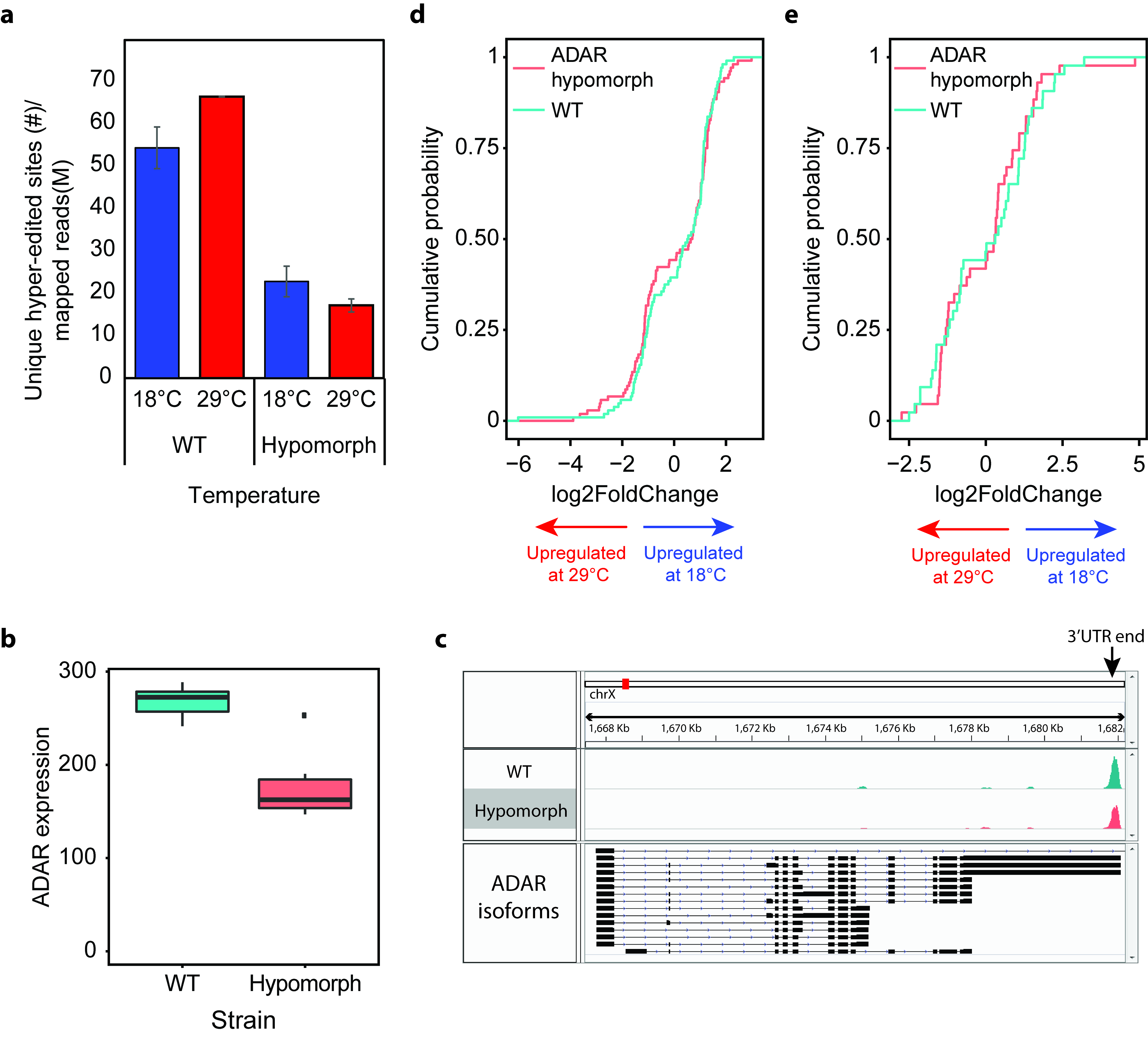

Supplement: S4 Fig — (A) The distribution of hyper-editing detected sites, normalized to the aligned number of reads, shows a different editing behavior for hypomorfic and WT flies. (B) ADAR hypomorph flies have lower expression of Adar mRNA comparing to WT flies. The data presents normalized read counts from 3' RNA-seq experiment (C) IGV snapshot of RNA-seq data from WT and ADAR hypomorph flies 3'. The single peak in both strains demonstrates no production of a new truncated protein in the ADAR hypomorph flies (D) Cumulative distribution plot for genes differentially expressed in 29°C vs. 18°C that are affected by ADAR hypomorph in both 18°C and 29°C. (E) Cumulative distribution plot for genes that are affected by ADAR hypomorph only in 29°C. For the comparisons presented in B and C Kolmogorov-Smirnov test showed that there are no significant differences between the curves (p = 0.83 and p = 0.69, respectively). (TIF) [file pgen.1006931.s004.tif]

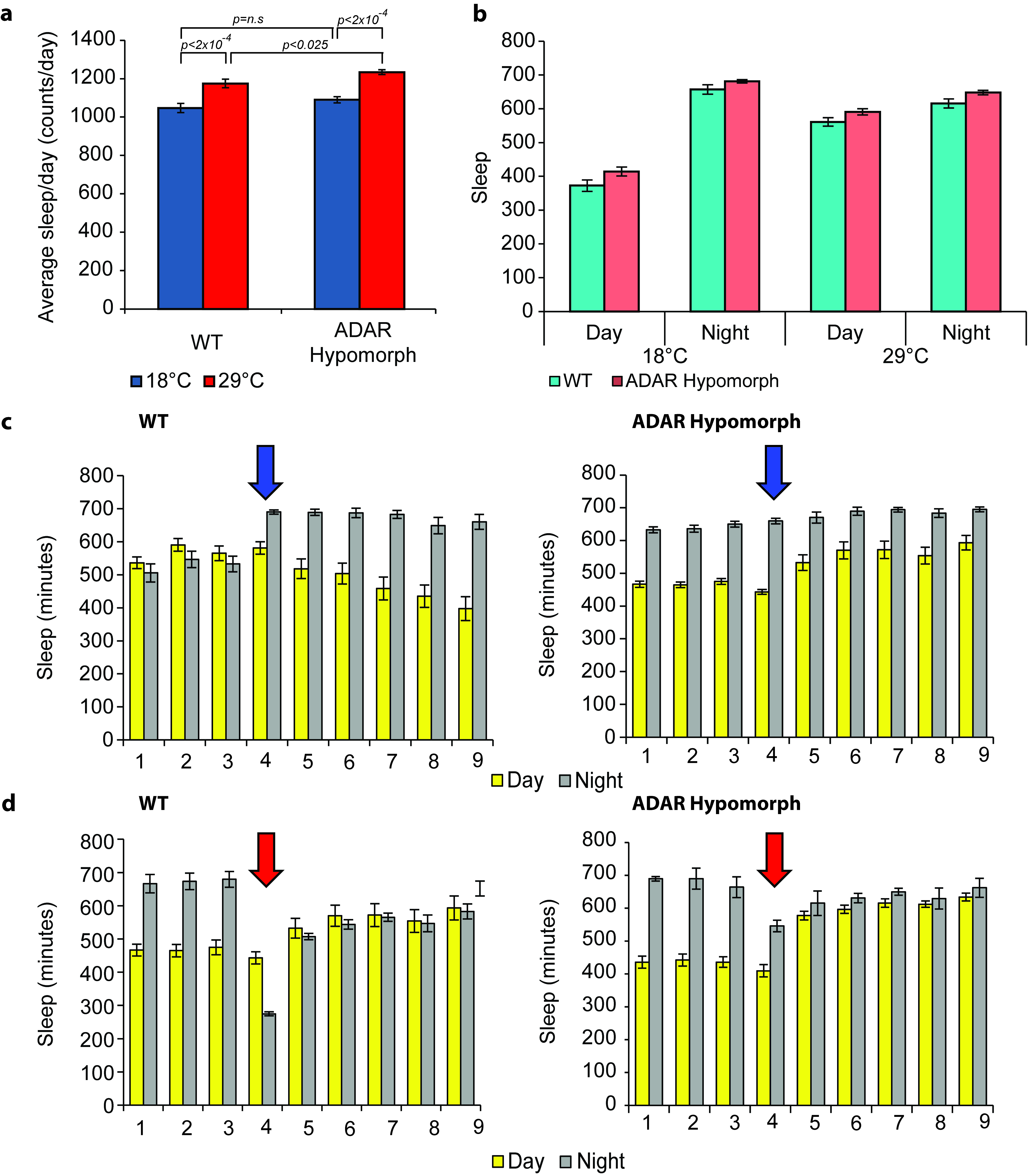

Supplement: S5 Fig — (A) No difference in average sleep between ADAR hypomorph and control flies, both at 18 and 29°C. (B) No difference in night or day sleep between ADAR hypomorph and control WT flies at 18 or 29°C. (C-D) Temperature transition (marked in an arrow) does not affect total sleep of ADAR hypomorph flies, in respect to their controls. (TIF) [file pgen.1006931.s005.tif]
